# Academic career preparation for residents - are we on the right track? Prevalence of specialized tracks in emergency medicine training programs

**DOI:** 10.1186/s12909-018-1288-x

**Published:** 2018-08-03

**Authors:** Jaime Jordan, Michael Hwang, Wendy C. Coates

**Affiliations:** 1 Department of Emergency Medicine, Harbor-UCLA Medical Center, 1000 W. Carson Street, Box 21, Torrance, CA 90502 USA; 2 David Geffen School of Medicine at University of California Los Angeles, Los Angeles, CA USA; 3 Los Angeles Biomedical Research Institute at Harbor-UCLA, Torrance, CA USA

**Keywords:** Career preparation, Graduate medical education, Academic medicine, Emergency medicine

## Abstract

**Background:**

Residency prepares trainees to deliver clinical care. It’s unknown if there is adequate career preparation, particularly for academic medicine. Prior literature has shown that interest in pursuing an academic career wanes during residency. Few trainees believe residency provides them with the necessary skills to be successful in academic medicine. Formalized areas of concentration may allow for deepened experience and mentorship in a specific field and may contribute to increased scholarly productivity which has been associated with selecting an academic career. Some training programs have instituted specialized tracks to allow residents to explore and develop an academic or clinical niche. The pervasiveness and characteristics of tracks currently available are unknown. A crucial first step in understanding how to best prepare residents for future careers is to understand current practice. The objective of this study was to identify the prevalence and characteristics of specialized tracks in emergency medicine (EM) training programs in the United States of America (USA).

**Methods:**

Allopathic EM training programs in the USA were identified by the Society for Academic Emergency Medicine residency catalogue. Program websites were identified through this catalogue and an internet search. Each page of a program’s website was dissected to identify basic program information and descriptions of their curricula including presence and descriptions of specialized tracks. Descriptive statistics are reported.

**Results:**

163 programs were identified, 156(95.7%) programs provided detailed descriptions of their curricula on their program website. 33/156(21.2%) offered dedicated tracks. Tracks were more common in four year programs (15/40;37.5%) compared to three years (18/116;15.5%). 23/33(70%) programs with tracks provided titles of their tracks and these commonly (20/23;87%) mirrored typical fellowships in EM. For programs that described the timing of tracks (15/33;45.5%), most spanned multiple years of training (12/15;80%).

**Conclusion:**

The presence of specialized tracks is not widespread in EM training programs in the USA, but is more commonly seen in four year programs. The timing of tracks varied but typically spanned multiple years of training. This information is a critical first step to allow future research to understand the impact of specialized tracks and their role in EM career choice and preparation for an academic career.

## Background

It is not well understood why some physicians pursue a career in academic medicine while others choose private practice. Multiple contributing factors have been identified in the literature, though at times with conflicting results, and include personal preferences such as family obligations, desired lifestyle, commitment to research, and financial considerations as well as environmental factors such as training program characteristics (format, size, region), mentorship, and resource availability [1–11]. While many residency applicants indicate a preference for “academics” during the interview process, interest in pursuing an academic career seems to wane as residents progress through training [5, 6, 12]. Limited data suggest that size and location of program may play a role in career choice [3]. It is possible that senior medical students have been exposed only to the academic lifestyle, sometimes continuously since Kindergarten, and therefore aspire to this due to a lack of exposure to other options.

In contrast, the central focus of residency is to prepare trainees to deliver clinical care to patients. While there is a requirement for “scholarly activity” for emergency medicine (EM) training programs in the United States of America (USA), as stipulated by the Accreditation Council on Graduate Medical Education (ACGME) there is great flexibility in how it is met [13]. It is reasonable to assume that all residency programs provide some sort of career counseling, but we wondered if there is widespread systematic career preparation for those who are suited to a career in academic medicine or other sub-specialty areas? Residents have noted inadequate exposure to research, mentorship, and training to prepare them for a successful academic career [2, 12]. In an effort to bridge this gap, some programs have implemented “scholarly tracks” or “selectives” with clear goals and objectives to provide additional training and allow residents to explore and develop a clinical or academic niche in EM [14]. Curricular tracks can increase residents’ scholarly productivity, which has been associated with selecting a career in academics [6, 7, 14–17]. It is unclear how prevalent track programs are and what value they provide in EM residency training, including impact on career choice and the decision to pursue an academic career. As a crucial first step to understand how to best prepare residents for their future careers and whether scholarly tracks are valuable in this pursuit, we must first explore the current practice. The objective of this study is to describe the prevalence and characteristics of scholarly tracks in allopathic EM residency programs within the USA.

## Methods

### Study design

We performed a systematic search of public residency program websites. In the USA, residency training programs typically have a program website to provide prospective applicants with information regarding the program’s faculty, curricular offerings, clinical environment, research, etc. This study was reviewed by the Institutional Review Board (IRB) of the Los Angeles Biomedical Research Institute at Harbor-UCLA Medical Center and given “exempt” status.

### Study protocol

We compiled a list of allopathic EM residency training programs listed in the Society for Academic Emergency Medicine (SAEM) residency directory, a searchable, comprehensive directory of all EM residency training programs in the USA that is commonly known to medical students and EM providers [18]. Individual program websites were identified through this directory. We extended our search to the internet to identify any rare program website that might not have been listed on the SAEM site. Each residency program website was accessed, and each page evaluated word for word by a single member of the study team during a 1 month period, July 1st, 2016 – July 31st, 2016.

### Measurements and key outcome measures

The study team agreed upon categorical outcomes a priori by discussion after a comprehensive literature review on career choice as well as available clinical and non-clinical post-graduate fellowships in EM. The latter search aimed to identify fellowships that were available for formal board certification by the American Board of Emergency Medicine (ABEM) as well as those typically offered as advanced skill development fellowships that do not have a route to board certification (i.e. education, simulation, etc.). For the purpose of this study, we considered a track to be an organized, longitudinal curricular component for residents to explore and/or gain experience in a specialized area or niche of emergency medicine. We defined longitudinal to be more than a single rotation in order to differentiate tracks from electives. We recorded demographic information pertaining to the residency program: (format; region – as categorized by the SAEM residency directory [18]; number of residents/year; medical school affiliation) and any available details of the particular curriculum of the tracks: (presence/absence; title; timing; duration; and description).

### Data analysis

Descriptive statistics are reported.

## Results

One hundred and sixty-three programs were identified, 156 (95.7%) programs provided detailed descriptions of their curricula on their program’s public website. 33/156 (21.2%) offered dedicated tracks. Tracks were present in 15/40 (37.5%) four-year programs compared to 18/116 (15.5%) three-year programs. Programs in the northeastern US more commonly had tracks (13/45; 28.9%) followed by programs in the midwest (8/36; 22.2%), west (4/21; 19%), southwest (3/16; 15.8%) and southeast (5/38; 13.2%). Programs with tracks had a mean number of residents per year of 12.75 compared to programs without tracks where the mean number of residents per year was 11.27. Most programs (150/156; 96.2%) were associated with a medical school. All programs with tracks were associated with a medical school. For programs in which the names of the tracks were identified (23/33; 70%), these commonly (20/23; 87%) mirrored typical fellowships in EM in the USA. Almost half of the programs specified the timing of tracks (15/33; 45.5%), and most of these spanned multiple years of training (12/15; 80%).

## Discussion

Scholarly tracks have the potential to allow trainees to focus their scholarly efforts, explore and develop an academic or clinical niche, and develop skills to better prepare them for their future careers under the guidance of dedicated mentors. Prior literature has suggested strategies for successful implementation of scholarly tracks in EM training programs [14], yet our study demonstrates that these types of offerings have yet to become widespread. Based on our experience as EM educators, we considered the possible barriers to implementation which could include lack of awareness or proven efficacy of these novel programs; resource limitations surrounding service needs of clinical departments; funding for salary support or tuition for formalized programs; compliance with ACGME clinical and didactic guidelines; or simply a lack of familiarity with this educational method. It is encouraging to note that most programs already have the fundamental resources needed –faculty with specialized expertise and interested residents. Since there are limited data demonstrating outcomes and impact of such tracks, training programs may be hesitant to implement them until research demonstrates favorable educational and career outcomes. We agree with Regan et al. who called for additional study into the various formats and impact of scholarly tracks on both scholarship and career choice [14].

In the USA, EM residency programs can be either 3 or 4 years in duration and are equivalent in terms of certifying trainees to be able to apply for specialty certification by ABEM. Individual programs and institutions decide which format best suits their needs and ability to meet the program training requirements. Tracks are more common in four-year programs which may allow more time and flexibility to integrate curricular content and supplemental activities outside of mandated program requirements. Lubavin et al. noted that graduates of four-year programs are more likely to choose a career in academics [9]. It is possible that these programs may have implemented tracks as a way to meet the preconceived needs of their residents who selected this format of training. Alternatively, one could posit that the existence of the tracks provided an introduction to academic EM to undecided residents who were exposed to them.

Interestingly, our study found that tracks were more common in programs in the northeast, which has also been associated with selection of an academic career in prior literature [3]. As in the case of the four-year program format, we cannot be sure whether it is the educational environment of the program that has successfully implemented scholarly tracks that provide further training and mentorship which contributes to resident selection of an academic career as others have suggested [5, 7, 19], or if residents who desire a career in academics self-select programs with these curricular characteristics.

In the USA, graduates of EM residency training programs can complete additional training in the form of fellowships which can be both clinical (i.e. critical care, pediatric EM, toxicology) or nonclinical (i.e. research, education, administration). We found that the types of tracks offered broadly mirrored available fellowships in EM. This supports one of the main goals of scholarly tracks, which is to allow trainees to develop a niche and prepare for their future careers. Tracks allow residents to explore areas of expertise without committing to additional training years. There are multiple potential benefits to providing this type of exposure during residency rather than after. Exposing trainees to a sub-specialty area enables them to make an informed decision of whether to pursue in-depth training through a fellowship. Should they decide on fellowship training, the work they have begun in residency serves as a foundation to embark on their fellowship at a higher skill level and to have the beginnings of a mentor network already in place. Ideally, the work done in the course of the tracks affords them the opportunity to demonstrate scholarly productivity, thus making them more competitive applicants in their job search.

Our study found that most scholarly tracks are longitudinal in nature, which aligns with a central goal of this method – career development. Many trainees continually evolve with respect to their preferences and skillsets as time progresses. A program that provides ongoing mentorship and flexibility to meet the evolving needs of the learner will be most impactful.

Our pilot study describes the prevalence and some common characteristics amongst scholarly tracks in EM residency training programs in the USA. This is a necessary first step to inform EM educators of the track model and consider how to best prepare trainees for their future careers. As a specialty, it is important to ensure that we can systematically recruit and effectively prepare those who will train future generations of EM physicians. Although this study is limited to the USA, future research should be expanded to include Canada (where EM training programs are well established and frequently include academic streams) and other countries that have EM training programs. While not all trainees who participate in the scholarly track educational system will eventually choose an academic career, each stands to gain a depth of understanding in one or more areas that could lead to improved patient care and clinical proficiency. Since these outcomes are currently not well studied, it is important to answer the many questions about the impact of scholarly tracks and future academic and clinical proficiency. A logical next step should focus on identifying and measuring objective outcomes and the impact of scholarly tracks, especially as they relate to career selection and preparation, as well as early scholarly productivity. It is likely that a qualitative study to compare outcomes of this method with alternative methods for career development in residency training could illuminate a quantitative research hypothesis to measure the impact of scholarly tracks in residency. Important information also includes details on curricular content, similarities and differences amongst programs, and barriers to implementation and maintenance along with advice to those who wish to start such a program.

### Limitations

Since our study was a review of publicly available information, we may have missed data on scholarly tracks that exist in programs but are not advertised on their websites. However, since prospective applicants often view program websites and use this information in deciding whether to apply to and rank a program, we assumed that most programs would have included the most current and complete information on their websites. Detailed description of the curricular content of tracks was not available and may vary amongst programs. Additionally, this study was of training programs in the USA and so our results may not be generalizable to other countries. However, we believe that our results may be of interest to educators globally as they consider career preparation opportunities for their trainees. Our study objective did not address the topics of characteristics of individual candidates or a quota of residents to be recruited to an academic career.

## Conclusion

The presence and structure of specialized tracks is not widespread in EM training programs, but is more commonly seen in programs that are the four-year format; of larger size; located in the northeast region; and were most commonly longitudinal in structure. This information is a critical first step to allow future research to understand the impact of specialized tracks and their role in EM career choice and preparation for an academic career.

## Abbreviations

ABEM: American Board of Emergency Medicine
ACGME: Accreditation Council for Graduate Medical Education
EM: Emergency medicine
IRB: Institutional Review Board
SAEM: Society for academic emergency medicine
USA: United States of America
